# The changing landscape of left ventricular assist device care in the setting of a pandemic

**DOI:** 10.1002/ehf2.12845

**Published:** 2020-07-07

**Authors:** Jennifer M. Amadio, Darshan H. Brahmbhatt, Heather J. Ross, Vivek Rao, Filio Billia

**Affiliations:** ^1^ Peter Munk Cardiac Center, Division of Cardiology University Health Network Toronto ON M5G 2N2 Canada; ^2^ National Heart and Lung Institute Imperial College London London UK; ^3^ Peter Munk Cardiac Center, Division of Cardiovascular Surgery University Health Network Toronto ON Canada

The spread of coronavirus disease 2019 (COVID‐19) throughout the globe has forced a change in how routine medical care is delivered. Patients with heart failure (HF) alongside those with other cardiovascular co‐morbidities are at increased risk of morbidity and mortality from COVID‐19.^1^, ^2^ In Mainland China, the case fatality rate for patients with cardiovascular disease was 10.5%, with 4.2% of cardiovascular disease cases comprising 22.7% of all deaths.^3^, ^4^ This has resulted in a move to reduce in‐person evaluation (IPE), as travel to hospitals and other healthcare facilities poses a risk to patients in contracting the disease. At the same time, because of increased inpatient workload, healthcare professionals are being redeployed from commitments in ambulatory settings to cope with patients admitted with COVID‐19, forcing cardiologists to increase the capacity of their virtual clinics to manage patients from afar.

As cardiologists, we have a large and successful experience in remote monitoring of HF patients using a range of technologies: from telephone consultation to implantable and wearable technologies.^5^, ^6^ However, remotely monitoring patients with end‐stage HF, treated with durable left ventricular assist devices (LVADs), may prove difficult. The mainstay of management includes (i) determination of fluid balance; (ii) frequent bloodwork to exclude anaemia from haemolysis or bleeding and adequacy of anticoagulation; and (iii) interrogation of device settings and optimization of pump speeds. There is a paucity of reports on how these patients can be managed from afar.^7^

Transitioning these patients to virtual care models has proven to be a challenge for the advanced HF community due to the more severe underlying HF condition. Moreover, these patients will wait longer for heart transplant, as programmes remain suspended for all but the highest risk patients. Here, we describe the transition of care for LVAD patients at the Toronto General Hospital in Ontario, Canada.

We moved a large volume of HF patients to virtual patient consultations using the Ontario Telehealth Network (OTN), a provincial platform for secure telephone and video consultation with patients. This follows a similar scheduling to IPE visits, where patients are given a fixed appointment time to present for review through telehealth medicine and videoconferencing. Interactions are recorded in a clinic letter, and medications can be prescribed by contacting the patients' pharmacy directly. While this can be useful for reviewing a patient's symptoms, it allows little examination other than inspection, unless there is coordination with a healthcare provider at the other site. However, if this is conducted between the physician and the patient at home, limited data are provided. It can also be difficult to track patient weight changes during single telehealth visits, without a means of recording a trend of changes and knowing which symptoms were associated with changes in weight. Fluid balance remains key to ensure adequate preload to prevent low‐volume left ventricular suction events.

To improve this, our team used the Medly program, an individualized, rules‐based, stand‐alone application that allows patients and clinicians to monitor patient's HF disease state. The use of Medly has been shown to reduce HF and all‐cause hospitalizations with improvement in HF‐related quality of life.^6^ The Medly program uses a mobile telephone application, a scale, and a blood pressure cuff to track each patient's weight, vital signs, and symptoms on a daily basis. Unlike a typical HF patient, LVAD patients are unable to record vital signs unless they have access to Doppler ultrasound monitoring for mean arterial pressure. Some of our LVAD patients do have access to a Doppler for this monitoring. LVAD patients also comment on symptoms of fever, driveline infection, new bleeding, or neurological symptoms. They are aware that if they develop any of these, they require urgent evaluation in a hospital setting. Cognitive and neurological evaluation are assessed during the OTN virtual consultation with patients and support persons. Based on the data, Medly provides specific real‐time patient self‐management alerts using a clinically validated algorithm.^6^, ^8^ These alerts are also forwarded to the patient's care team for review by a nurse practitioner and the primary physician. Decisions are made by the care team to adjust medications, act on symptoms, or trigger IPE if acute decompensation is suspected. The goal is to help patients stay well, prevent HF exacerbations, and allow patients to remain out of hospital.

Our novel solution is to combine remote monitoring data from Medly with virtual patient consultations (through OTN) to optimize clinical care and reduce the number of LVAD patients requiring ambulatory visits to the Toronto General Hospital. This has helped better assess patients' symptoms (such as orthopnoea, paroxysmal nocturnal dyspnoea, or peripheral oedema) and maintain a thorough assessment of fluid balance, allowing early intervention with the reinforcement of lifestyle advice on fluid and sodium restriction and alteration in diuretic dosing.^6^

Our patient population includes 35 patients treated with a range of devices, including HeartMate™ II Left Ventricular Assist System (Abbott; Chicago, IL, USA), HeartMate™ 3 (Abbott), and the HeartWare™ HVAD™ (Medtronic; Minneapolis, MN, USA). Initially, we planned to transition patients with destination therapy LVAD to telemonitoring visits. With COVID‐19 numbers rising, we have now enrolled all LVAD patients for remote non‐invasive monitoring, including bridge to transplant and bridge to candidacy patients who are typically seen on a monthly basis (*Table* *1*). The care of these patients now includes a scheduled virtual clinical encounter and tracking their disease trajectory daily using Medly remote monitoring. Through this process, we aim to intervene earlier to prevent HF decompensation and hospital admission.

**Table 1:** Patient care schedule for Toronto General Hospital LVAD programme

LVAD patient population	Routine care schedule (before COVID‐19 outbreak)	Revised care schedule (since COVID‐19 outbreak)
Destination therapy	1. Quarterly in‐person evaluation with echocardiogram	1. Quarterly virtual patient consultation 2. Echocardiography deferred
Bridge to candidacy	1. Monthly in‐person evaluation 2. Echocardiogram every 3 months	1. Monthly virtual patient consultation 2. Enrolment onto Medly program
Bridge to transplant	1. Monthly in‐person evaluation 2. Echocardiogram every 3 months	1. Monthly virtual patient consultation 2. Enrolment onto Medly program
Recent LVAD implantation (first month)	1. Weekly in‐person evaluation	1. Weekly in‐person evaluation 2. Enrolment onto Medly program

COVID‐19, coronavirus disease 2019; LVAD, left ventricular assist device.

These monitoring data are combined with routine laboratory tests to monitor blood counts, electrolytes, creatinine, and international normalized ratio. Traditionally, we perform echocardiograms every three months to reassess heart function, aortic valve disease, LVAD cannula flows, optimize pump speed, and evaluate for complications like right ventricular failure or presence of intracardiac clots (at cannula site or otherwise). We have delayed these assessments during the peak of COVID‐19 to avoid unnecessary travel in stable patients.

There are patient limitations to remote monitoring. They require ability to operate a computer for OTN visits or operate a telephone for phone visits. They must be able to operate the Medly application on their cellular phone or tablet. Primarily, these individuals must be English speaking or have access to a translator to be able to communicate remotely with the healthcare team and to understand the management ‘prompts’ from Medly. All of our LVAD patients have a support person able to assist them during this time of remote monitoring, which can be particularly helpful should the patient have any long‐term cognitive deficits from a previous stroke, which has a higher prevalence in this population of HF patients.

Further limitations of remote management of patients with LVADs remain the interrogation of the device to ascertain the cause of any alarms, or device dysfunction, which can only be conducted in person. This is very different to most cardiovascular implantable electronic devices, such as pacemakers and implantable cardioverter defibrillators, which can be securely reviewed and interrogated remotely through dedicated paired radio frequency or Bluetooth transmitters. Despite the two largest manufacturers in the LVAD market having their own proprietary monitoring networks (Medtronic CareLink™ and Abbott Merlin.net™), these technologies are not available to HF patients with LVAD. Nationally, a minority of patients currently utilize remote monitoring of cardiovascular implantable electronic devices.^9^ This prevents the integration of physiological trends, such as intrathoracic impedance and an objective measure of daily activity, being used as part of the patient assessment. While it is unlikely that there will be a solution to this during the current pandemic, this should be an area for technological innovation for the management of patients with advanced HF, as the use of LVADs continues to increase.

Even with the benefits of telemonitoring, LVAD patients with acute decompensation, active bleeding, syncope, or other concerning symptoms will still require IPE and potential hospital admission. However, we hope that through daily monitoring with Medly, many complications may be avoided. Diuretic medications may be titrated from home, and fever or infections may be managed as an outpatient by ordering blood cultures at an outpatient laboratory if the patient is otherwise clinically stable. We are exploring the ability to incorporate questions into the Medly algorithm for these LVAD‐specific symptoms. Another possibility is to combine our remote monitoring data with direct haemodynamic monitoring for LVAD patients using the CardioMEMS HF System (Abbott Vascular), which has been shown to improve HF hospitalizations in patients with New York Heart Association Class III symptoms over 18 months of follow‐up.^5^, ^10^ At Toronto General Hospital, we currently do not have any LVAD patients with a CardioMEMS implantable device.

Ultimately, the implementation of remote monitoring allows us to provide accelerated care to our LVAD patients in this time of global pandemic. However, a limitation of this protocol is that we do not yet know if there will be any adverse effects or events related to providing virtual care. Prior to the pandemic, we were following five LVAD patients with Medly with no adverse events noted.

In times of crisis and stress on an already overburdened medical system, innovative solutions are required to continue to provide high‐quality care for patients. Tracking LVAD patients using remote monitoring systems and following objective data may help to guide our management and prevent unnecessary HF decompensation and visits to hospital.

## Conflict of interest

None.

## Funding

None.
